# Cooking and In Vitro Digestion Modulate the Anti-Diabetic Properties of Red-Skinned Onion and Dark Purple Eggplant Phenolic Compounds

**DOI:** 10.3390/foods11050689

**Published:** 2022-02-25

**Authors:** Alice Cattivelli, Angela Conte, Serena Martini, Davide Tagliazucchi

**Affiliations:** Department of Life Sciences, University of Modena and Reggio Emilia, Via Amendola 2-Pad. Besta, 42100 Reggio Emilia, Italy; alice.cattivelli@unimore.it (A.C.); serena.martini@unimore.it (S.M.); davide.tagliazucchi@unimore.it (D.T.)

**Keywords:** type 2 diabetes, polyphenols, food processing, mass spectrometry, in vitro digestion, thermal treatments

## Abstract

The intake of phenolic-rich foods is an emerging preventive approach for the management of type 2 diabetes, thanks to the ability of these compounds to inhibit some key metabolic enzymes. In this study, the influence of cooking and in vitro digestion on the α-glucosidase, α-amylase, and dipeptidyl-peptidase IV (DPP-IV) inhibitory activity of red-skinned onion (RSO) and dark purple eggplant (DPE) phenolic fractions was assessed. The applied cooking procedures had different influences on the total and individual phenolic compounds gastrointestinal bioaccessibility. DPE in vitro digested phenolic fractions displayed no inhibitory activity versus α-amylase and DPP-IV, whereas the fried DPE sample exhibited moderate inhibitory activity against α-glucosidase. This sample mainly contained hydroxycinnamic acid amides that can be responsible for the observed effect. Contrariwise, raw and cooked in vitro digested RSO phenolic fractions inhibited all three enzymes but with different effectiveness. Fried and raw RSO samples were the most active against them. Statistical analysis pointed out that quercetin mono-hexosides (mainly quercetin-4′-*O*-hexoside) were responsible for the inhibition of α-glucosidase, whereas quercetin di-hexosides (mainly quercetin-3-*O*-hexoside-4′-*O*-hexoside) were responsible for the DPP-IV-inhibitory activity of RSO samples. An accurate design of the cooking methods could be essential to maximize the release of individual phenolic compounds and the related bioactivities.

## 1. Introduction

Type 2 diabetes (T2D) is a primary reason of death and was estimated to have caused around 4 million deaths worldwide in 2017 [1]. Both T2D morbidity and mortality are increasing from year to year, affecting dramatically the health-care systems and quality of life [2,3,4]. This metabolic condition, characterized by elevated levels of blood glucose, can lead to development of chronic diseases affecting the cardiovascular, renal, and nervous systems [5,6,7,8]. Nowadays, the management of T2D aims to reduce blood glucose levels through both pharmacological and non-pharmacological (healthy lifestyle and balanced diet) treatments. To overcome the high costs and side effects of drug therapies, dietary supplementation with natural anti-diabetic agents is becoming increasingly crucial [9,10,11,12]. A plethora of researches have indicated that the intake of functional foods could help to prevent and moderate metabolic syndrome and T2D [13,14]. 

Phenolic compounds are secondary metabolites of plant origin that exert several beneficial effects on human health. They may act as natural antioxidants with anti-aging properties, are also able to decrease the risk of the onset of cardiovascular diseases and cancers, and also play a significant role against diabetes [13,15]. Phenolic compounds may exert their anti-diabetic activity, acting at different levels [15,16]. For example, polyphenols can reduce the absorption of dietary carbohydrates, ameliorate the function of β-cells, stimulate insulin secretion, and induce satiety [17]. They have shown to decrease post-prandial hyperglycemia by inhibiting the activity of amylolytic enzymes and preventing the hydrolysis of incretins through the inhibition of dipeptidyl-peptidase IV [18,19,20]. 

The enzymes α-amylase and α-glucosidase are involved in the gastrointestinal digestion of dietary carbohydrates to free monosaccharides. The enzyme α-amylase is located in the intestinal fluid and is able to cleave polysaccharides (such as starch and glycogen), releasing shorter oligosaccharides (dextrins, maltotriose, and maltose). In contrast, α-glucosidase is a digestive enzyme situated at the intestinal brush border that completes the hydrolysis of short oligosaccharides, releasing free monosaccharides, which are in turn absorbed by intestinal enterocytes. Various studies have demonstrated that α-amylase and α-glucosidase inhibitors from traditionally used plants can retard the digestion and absorption of complex polysaccharides, thus diminishing the post-prandial blood glucose level [21]. Oboh et al. [11] demonstrated the anti-diabetic effect of phenolic-rich aqueous extracts of purple and white onion and garlic. Similarly, Paoutsis et al. [22] reported high inhibitory effects of citrus, pomegranate, berries, and *Prunus* phenolic-rich extracts on α-amylase and α-glucosidase. Kwon et al. [10] indicated the positive effect of phenolic-rich extracts from two types of eggplant on hyperglycemia risk factors.

Dipeptidyl-peptidase IV (DPP-IV) is a serine protease placed in the brush-border membrane of intestinal enterocytes and responsible for the rapid inactivation of incretins hormones such as glucagon-like peptide 1 (GLP-1) and glucose-dependent insulinotropic polypeptide (GIP) [18]. Incretins are able to lower the hematic levels of glucose by stimulating the release of insulin and inhibiting the release of glucagon [23]. As a consequence, DPP-IV inhibition may lead to a reduction of hyperglycemia by improving insulin secretion [24]. Although there are no in vivo studies, several in vitro researchers highlighted the role of phenolic compounds as DPP-IV inhibitors [20,25].

Most of the studies about the inhibitory effect of phenolic compounds on key enzymes involved in the regulation of blood glucose levels have been carried out on pure food extracts [10,11,18,19,22]. However, this is far from the real conditions of food consumption and phenolic compounds release in the gastrointestinal tract. Indeed, some vegetable foods, such as eggplant and onion, are mainly consumed after cooking. Several previous studies have suggested that cooking treatments play a significant role in modulating the phenolic compounds content of foods [26,27,28]. The effects of thermal processes on the stability of phenolic compounds are manifold. Some compounds may be degraded by high temperatures (e.g., anthocyanins) or released in the cooking medium (e.g., flavonols during boiling), while others may increase their extractability through the matrix softening effect [29,30]. This depends on several factors such as the food matrix, the phenolic structure, and technological parameters: among others, cooking time, temperature, and medium [30]. 

Another aspect to be considered is that phenolic compounds, to exert their anti-diabetic effects by inhibiting intestinal enzymes, should be bioaccessible, i.e., they should be released from the food material and stable over the gastrointestinal transit [26]. Testing the anti-diabetic properties of digested samples is pivotal, since solely the phenolic compounds released during digestion may interact with the enzymes present in the intestinal tract. To the best of our knowledge, no studies have been published on the anti-diabetic properties of vegetable food after cooking and in vitro gastrointestinal digestion. 

Therefore, this study was designed to explore the in vitro anti-diabetic properties (i.e., inhibitory activity toward three key metabolic enzymes, DPP-IV, α-glucosidase, and α-amylase) of two vegetable matrices rich in phenolic compounds, dark purple eggplant (DPE) and red-skinned onion (RSO), subjected first to four different cooking processes (baking, boiling, frying, and grilling) and then to in vitro gastrointestinal digestion. 

## 2. Materials and Methods

### 2.1. Materials

Chemicals and enzymes for in vitro digestion, α-glucosidase, α-amylase, and DPP-IV assays were obtained from Sigma-Aldrich (Milan, Italy). Solvents for phenolic extraction and MS/MS were supplied by Bio-Rad (Hercules, CA, USA). C18 columns for solid-phase extraction (500 mg, 50 μm, 60 Å) were purchased from Incofar (Modena, Italy). Dark purple eggplant and red-skinned onion samples were purchased in a local supermarket.

### 2.2. Cooking Treatments and In Vitro Gastrointestinal Digestion

Dark purple eggplant and red-skinned onion samples were subjected to four different types of cooking: boiling, baking, frying, and grilling, as described in Martini et al. [27] and Cattivelli et al. [26]. Each cooking procedure was carried out in triplicate. For the in vitro gastrointestinal digestion procedure, the method elaborated in the framework of the COST Action INFOGEST was applied [31]. As reported in Martini et al. [27] and Cattivelli et al. [26], raw and cooked dark purple eggplant and red-skinned onion samples were subjected to three consecutive passages: oral phase, gastric phase, and intestinal phase. At the end of the intestinal phase, an aliquot of sample was withdrawn and stored at −80 °C for further analysis. All the digestions were carried out in triplicate.

### 2.3. Preparation of the Phenolic-Rich Fractions

For the phenolic compounds extraction, C18 columns were pre-conditioned by passing 4 mL of methanol (acidified with 0.1% formic acid) and subsequently 5 mL of water (acidified with 0.1% formic acid). After column conditioning, 2 mL of in vitro digested samples were loaded at the top the column. The first elution step was performed with 3 mL of water (acidified with 0.1% formic acid) in order to remove the unbound material. Subsequently, phenolic compounds were detached from the column by eluting with 3 mL of acidified methanol (0.1% formic acid). Each sample was extracted in triplicate. After methanol removal, samples were re-suspended in distilled water and stored at −80 °C for further analysis.

### 2.4. Characterization of the Phenolics Profile of Phenolic-Rich Fractions by Liquid Chromatography Electrospray Ionization Ion Trap Mass Spectrometer (LC-ESI-IT-MS)

Individual phenolic compounds present in the different phenolic-rich fractions were identified and quantified through liquid chromatography-mass spectrometry analysis. The instrumental characteristics, the liquid chromatography parameters, and the ESI-MS (both positive and negative) conditions were the same as previously detailed [32,33]. Specific standard compounds were used for phenolic compounds quantification as previously reported [26,27,32,33]. 

### 2.5. Biological Activity Analysis

#### 2.5.1. Measurements of α-Amylase Inhibitory Activity

The inhibitory activity of phenolic-rich fractions against α-amylase was established as described by Li et al. [34] with slight modifications. Briefly, 5 µL of α-amylase from porcine pancreas (2 U/mL) were combined with 45 µL of different concentrations of phenolic-rich fractions (5–200 μmol/L). After 20 min of incubation at 37 °C, 50 μL of 1% starch solution, prepared in 0.1 mol/L sodium phosphate buffer (pH 6.9 and containing 6.7 mmol/L of NaCl), were added and the reaction mix was further incubated for 10 min at 37 °C. After incubation, 50 µL of dinitrosalicylic acid solution were added to the mix, and samples were boiled for 15 min in water. Before reading, 450 µL of distilled water were added to the mixture, and 200 µL of each experiment were transferred to a 96-well plate and read at 540 nm using a microplate reader. The concentration of phenolic compounds required to inhibit α-amylase activity by 50% (IC_50_) was calculated by correlating the percentage of α-amylase inhibition with the phenolic compounds concentration (base-10 logarithm). The concentration of phenolic compounds was calculated by LC-ESI-IT MS/MS analysis. Values were expressed as μmol of phenolic compounds per mL. Analyses were carried out in triplicate. 

#### 2.5.2. Measurements of α-Glucosidase Inhibitory Activity

The α-glucosidase assay was carried out as stated in Bellesia et al. [35] adapted to a microplate reader. For the inhibition assay, 66.7 µL of 0.1 mol/L potassium phosphate buffer (pH 6.8), 20 µL of different concentrations of phenolic-rich fractions (5–200 μmol/L), 3.3 µL of reduced glutathione (3.3 mmol/L), and 5 µL of yeast α-glucosidase solution (0.2 U/mL) were mixed in a 96-well plate and pre-incubated for 20 min at 37 °C. Then, 5 µL of 5 mmol/L *p*-nitrophenyl-glucose substrate (diluted in potassium phosphate buffer 0.1 mol/L, pH 6.8) were added to start the reaction, and the plate was further incubated (20 min; 37 °C). Finally, the reaction was stopped by adding 150 µL of 1 mol/L sodium carbonate. Then, the plate was read at 405 nm. The concentration of phenolic compounds required to inhibit α-glucosidase activity by 50% (IC_50_) was calculated as reported in Section 2.5.1. Values were expressed as μmol of phenolic compounds per mL. As reported previously, three analytical replicates for each sample were performed.

#### 2.5.3. Measurements of Dipeptidyl Peptidase IV (DPP-IV) Inhibitory Activity

The DPP-IV enzyme was extracted from rat intestinal acetone powder as reported in the protocol of Tagliazucchi et al. [36]. For the inhibition assay, 80 µL of different concentrations of phenolic-rich fractions (5–200 μmol/L), 205 µL of Tris-HCl buffer (0.1 mol/L; pH 8), and 10 µL of extracted enzyme (0.1 U/mL in the assay) were added to a 96-well plate and pre-incubated for 20 min at 37 °C. Then, the reaction was started by the addition of 5 µL of 6.4 mmol/L Gly-Pro-*p*NA. The quantity of released *p*-nitroanilide (*p*NA) was determined following 20 min of reaction at 37 °C by reading at 405 nm with a microplate reader. As reported above, the IC_50_ values were determined as reported in Section 2.5.1. Values were expressed as μmol of phenolic compounds per mL. For the enzymatic assay, three analytical replicates for each sample were carried out.

### 2.6. Statistics

Data are shown as mean ± standard deviation for three replicates for each sample. Univariate analysis of variance (ANOVA) coupled to Tukey’s post hoc test was executed using Graph Pad prism 6.0 (GraphPad Software, San Diego, CA, USA.). The differences were evaluated significant when *p* < 0.05. Principal component analysis (PCA) was performed using Solo software (Eigenvector Research Inc., Manson, WA, USA), and data are presented as biplots. Before performing PCA, the mean data were normalized to neutralize the influence of any hidden factors. Correlation and non-linear regression analysis were carried out with Graph Pad Prism. The correlation, expressed as Pearson *r*-value, was considered significant when *p* < 0.05.

## 3. Results and Discussion

### 3.1. Phenolic Compounds Profile of Phenolic-Rich Fractions Extracted from Raw and Cooked In Vitro Digested Dark Purple Eggplant (DPE) 

A total of 12, 15, 9, 14, and 20 individual phenolic compounds were identified and quantified in the phenolic-rich fractions of in vitro digested raw, baked, boiled, fried, and grilled DPE, respectively. The list of detected compounds, together with the quantitative information, is given in Table 1. From a quantitative point of view, in in vitro digested raw DPE phenolic-rich fraction, hydroxycinnamic acids made up the majority of the compounds present (96.7% of total phenolic compounds), while flavonols represented the remaining 3.3%. The most abundant compounds in the in vitro digested raw DPE phenolic-rich fraction were 5-*O*-caffeoylquinic acid *trans* and the oxidation products caffeoylquinic acid dehydrodymer isomers. The total amount of bioaccessible phenolic compounds quantified by mass spectrometry in in vitro digested raw DPE phenolic-rich fraction was 10.74 ± 0.25 μmol/100 g.

As shown in Table 1, all heat treatments resulted in an increase in bioaccessible total phenolic compounds compared to the in vitro digested raw phenolic-rich fraction. Frying accounted for the largest increase in total phenolic compounds (2674.2%), which was followed by grilling (1516.7%), baking (764.71%) and boiling (247.5%). These results suggested that cooking is one of the key aspects in the release of phenolic compounds from the food matrix during digestion, as already stated by other authors [27,37,38,39]. This cooking-related impact may be a consequence of the matrix-softening effect promoted by the cooking methods that enhance the release and consequently phenolic compounds bioaccessibility [30,37]. As previously reported, distinct thermal treatments may have a different effect on the bioaccessibility of phenolic compounds [26,27,38,39,40,41].

**Figure 1:**
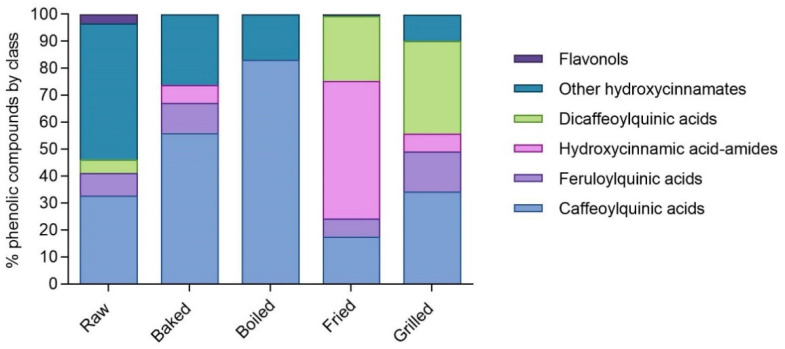
Percentage distribution of the main classes of phenolic compounds in raw and cooked in vitro digested dark purple eggplant phenolic-rich fractions. The percentage values are referred to the total concentration of phenolic compounds identified in the raw and cooked in vitro digested dark purple eggplant samples as reported in Table 1 and expressed as μmol of compound/100 g of raw or cooked eggplant.

The applied cooking methods also allowed a distinctive release in specific phenolic compounds resulting in a different phenolic profile in the in vitro digested phenolic-rich fractions (Table 1). Grilled, fried, and baked in vitro digested DPE phenolic-rich fractions showed the highest amount of caffeoylquinic acids, especially in the compounds 5-*O*-caffeoylquinic acid *trans* and *cis*. In addition, fried and grilled phenolic-rich fraction samples also displayed the highest amount of bioaccessible dicaffeoylquinic and feruloylquinic acids. Finally, the fried sample phenolic profile was dominated by hydroxycinnamic acid amides. The percentage of distribution of the different phenolic compounds grouped by class is shown in Figure 1. Caffeoylquinic acids represented the most important class of phenolic compounds in boiled (83.1% of total phenolic compounds) and baked (56.0% of total phenolic compounds) in vitro digested DPE phenolic-rich fractions. The grilled in vitro digested DPE phenolic-rich fraction was mainly composed of dicaffeoylquinic acids (34.4% of total phenolic compounds) and caffeoylquinic acids (34.2% of total phenolic compounds), whereas hydroxycinnamic acid amides (51.2% of total phenolic compounds) were predominant in the fried sample followed by dicaffeoylquinic acids (24.0% of total phenolic compounds).

### 3.2. Phenolic Compounds Profile of Phenolic-Rich Fractions Extracted from Raw and Cooked In Vitro Digested Red-Skinned Onion (RSO)

As reported in Table 2, the number of phenolic compounds identified in raw, baked, boiled, fried, and grilled in vitro digested RSO phenolic rich-fractions was 15, 10, 6, 9, and 11, respectively. In in vitro digested raw RSO phenolic rich-fraction, the class of phenolic compounds most commonly present was flavonols (10 compounds) followed by anthocyanins (3 compounds) and dihydro-flavonols (one compound). In the in vitro digested raw RSO sample, the quantitative phenolic profile was dominated by flavonols and in particular by quercetin mono-hexosides and quercetin di-hexosides. The total concentration of bioaccessible phenolic compounds quantified by mass spectrometry in the in vitro digested raw RSO phenolic-rich fraction was 85.49 ± 0.39 μmol/100g of onion. As reported in Table 2, heat treatments also caused an increase in bioaccessible total phenolic compounds in the in vitro digested phenolic-rich fractions of cooked RSO with respect to the raw sample, although it was less evident than in DPE. The greatest increase occurred with grilling treatment (95.5%), which was followed by baking (12.5%), boiling (2.4%), and frying (1.9%). A comparable trend was noted for the content of total flavonols as a function of the cooking treatment. The greatest augment in total bioaccessible flavonols content, compared to the raw sample, was found after grilling (391%), which was followed by baking (19.2%) and frying (8.5%). Boiling, on the other hand, is the only cooking procedure that brought about a reduction in the bioaccessible total flavonols content (43.6%) in comparison with the raw sample. This behavior after the different cooking methods and in vitro digestion was already reported by other studies. Specifically, the authors found that baking, grilling, and frying enhanced the bioaccessibility of flavonols in green pepper, cactus cladodes, and onion, whereas the flavonols content decreased after boiling treatment [26,38,41]. Considering the individual phenolic compounds, the one with the greatest bioaccessibility in all of the samples was quercetin-3-*O*-hexoside-4′-*O*-hexoside followed by quercetin-4′-*O*-hexoside (Table 2). In addition, grilling was the only treatment able to preserve anthocyanins after in vitro digestion. All other treatments caused a complete loss of these compounds in in vitro digested RSO phenolic-rich fractions (Table 2). 

**Figure 2:**
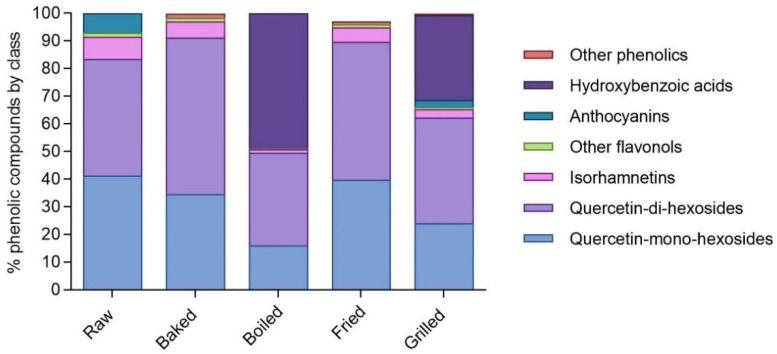
Percentage distribution of the main classes of phenolic compounds in raw and cooked in vitro digested red-skinned onion phenolic-rich fractions. The percentage values are referred to the total concentration of phenolic compounds identified in the raw and cooked in vitro digested red-skinned onion samples as reported in Table 2 and expressed as μmol of compound/100 g of raw or cooked onion.

Additionally in RSO, the different cooking methods modified not only quantitatively but also qualitatively the phenolic profile after in vitro digestion, as already stated for DPE (Figure 2). Raw, baked, and fried in vitro digested RSO phenolic-rich fractions contained mainly quercetin-mono hexosides (41.2%, 34.6%, and 39.7% of total phenolic compounds, respectively) and quercetin-di-hexosides (42.3%, 56.6%, and 50.0% of total phenolic compounds, respectively). On the contrary, the predominant compounds in boiled in vitro digested RSO phenolic-rich fraction were hydroxybenzoic acids (48.9% of total phenolic compounds).

### 3.3. Inhibitory Effect of In Vitro Digested Phenolic Rich-Fractions on α-Amylase, α-Glucosidase, and Dipeptidyl Peptidase-IV (DPP-IV) Activities

In vitro digested phenolic-rich fractions of DPE and RSO were tested for their capacity to inhibit α-amylase, α-glucosidase, and DPP-IV, which are the three key enzymes considered the pharmacological targets in the anti-diabetic therapy. The in vitro digested phenolic-rich fractions of DPE displayed no inhibitory effect versus α-amylase and DPP-IV enzymes, independently of the cooking treatment. The only exception was the in vitro digested fried DPE sample, which showed moderate inhibitory activity toward the enzyme α-glucosidase with an IC_50_ value of 33.28 ± 1.02 µmol of phenolic compounds/L. 

Differently from the others, an in vitro digested DPE fried sample contained predominantly hydroxycinnamic acid-amides (51.2% of total phenolic compounds, Figure 1) and especially N^1^,N^5^-di-dihydrocaffeoyl-spermidine and N^1^,N^10^-dihydrocaffeoyl-caffeoyl-spermidine (49.9% of total phenolic compounds in the sample). The other two representative classes of phenolic compounds in the in vitro digested DPE fried sample were dicaffeoylquinic acids and caffeoylquinic acids. Previous studies showed that caffeoylquinic acids did not present α-glucosidase inhibitory activity [42,43,44]. Indeed, boiled and baked samples contained a high percentage of caffeoylquinic acids and were not active against α-glucosidase. Contrariwise, dicaffeoylquinic acids were found to be able to inhibit α-glucosidase but with IC_50_ values in the order of hundreds of μmol/L [42,43,44]. Moreover, the in vitro digested grilled sample had a higher percentage of dicaffeoylquinic acids compared to the fried sample but no inhibitory activity. Therefore, hydroxycinnamic acid-amides can be considered the α-glucosidase-inhibitory active compounds present in the in vitro digested fried sample. Furthermore, there is strong evidence that hydroxycinnamic acid spermidine derivatives and, more in general, phenol amides exhibit a broad range of bioactivities including antioxidant, anti-microbial, and anti-cancer activities as well as protective effects against cardiovascular and metabolic syndrome-related diseases [45].

**Figure 3:**
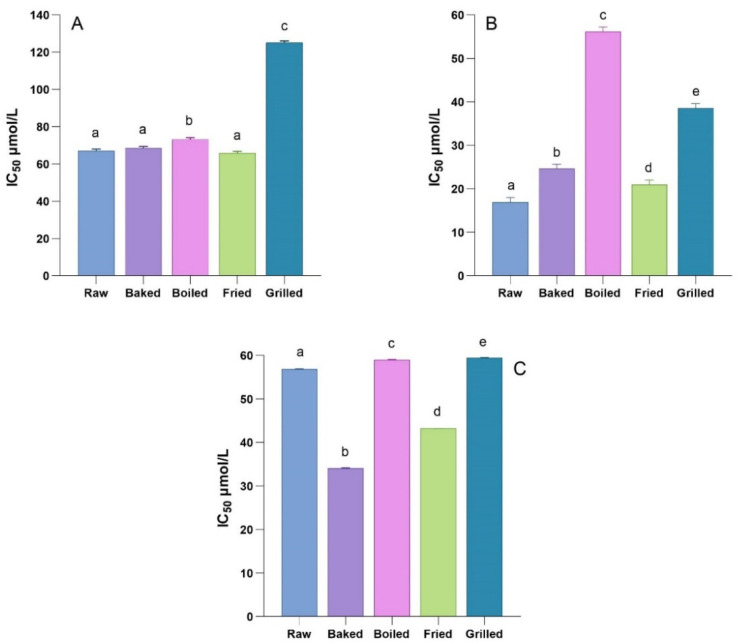
Effect of in vitro digested red-skinned onion phenolic-rich fractions on the activity of key metabolic enzymes. (**A**) Inhibitory activity against α-amylase. (**B**) Inhibitory activity against α-glucosidase. (**C**) Inhibitory activity against dipeptidyl-peptidase IV. The concentration of phenolic compounds (expressed as μmol of phenolic compounds per mL) required to inhibit the enzymatic activity by 50% (IC50) was calculated by plotting the percentage of the enzyme inhibition with the phenolic compounds concentration (base-10 logarithm). The concentration of phenolic compounds was determined by LC-ESI-IT MS/MS analysis and detailed in Table 2. Different letters mean significant different (*p* < 0.05) values

As reported in Figure 3, the phenolic-rich fractions of in vitro digested RSO were able to inhibit all three key enzymes. Samples that showed the greatest inhibitory activity against the α-amylase enzyme (Figure 3A) were fried, raw, and baked in vitro digested RSO samples (IC_50_ = 65.76 ± 1.00 µmol/L, 66.98 ± 1.00 µmol/L and 68.46 ± 1.01 µmol/L, respectively). No significant differences (*p* > 0.05) were found between the α-amylase-inhibitory activity of these samples. The in vitro digested boiled sample differed slightly but significantly (*p* < 0.05) from raw, baked, and fried in vitro digested RSO samples (IC_50_ = 73.15 ± 1.00 µmol/L), while the in vitro digested grilled sample showed the lowest inhibitory activity (IC_50_ = 125.10 ± 1.02 µmol/L). 

As shown in Figure 3B, the RSO samples differed in their inhibitory activity toward the α-glucosidase enzyme. In this case, the in vitro digested raw RSO sample was the most active (IC_50_ = 16.93 ± 1.09 µmol/L), which was followed by the in vitro digested fried, baked, grilled, and, lastly, boiled sample (IC_50_ = 20.92 ± 1.07 µmol/L, 24.59 ± 1.05 µmol/L, 38.54 ± 1.03 µmol/L, and 56.15 ± 1.08 µmol/L, respectively). 

Although there are no studies on cooked and digested samples, the α-glucosidase and α-amylase inhibitory effects of red-skinned onion extracts have been confirmed by other researchers [11,46,47]. Accordingly, in all these studies, the red-skinned onion extracts were most active against α-glucosidase with respect to α-amylase. The inhibitory effects of the red-skinned onion extracts versus α-glucosidase and α-amylase have been assigned to the occurrence of phenolic compounds and in particular flavonols and anthocyanins, which displayed enzymes inhibitory activity [19,48,49]. 

As depicted in Figure 3C, all of the in vitro digested RSO phenolic-rich fractions were able to inhibit DPP-IV activity with grilled and fried samples showing the highest inhibitory activity (IC_50_ = 34.14 ± 1.05 and 43.23 ± 1.04 µmol/L, respectively). A significantly lower amount of activity was observed for the in vitro digested raw sample (IC_50_ = 56.85 ± 1.05 µmol/L) as well as for boiled and grilled samples (IC_50_ = 59.01 ± 1.10 and 59.45 ± 1.03 µmol/L, respectively). To the best of our knowledge, there are no studies in the literature on the DPP-IV-inhibitory activity of red-skinned onion extracts. Nevertheless, Kim et al. demonstrated that some flavonol glycosides were potent DPP-IV inhibitors [50]. Therefore, it can be speculated that flavonol glycosides are the active compounds in red-skinned onion in vitro digested samples able to inhibit DPP-IV activity.

### 3.4. Statistical Analysis

#### 3.4.1. Principal Component Analysis

**Figure 4:**
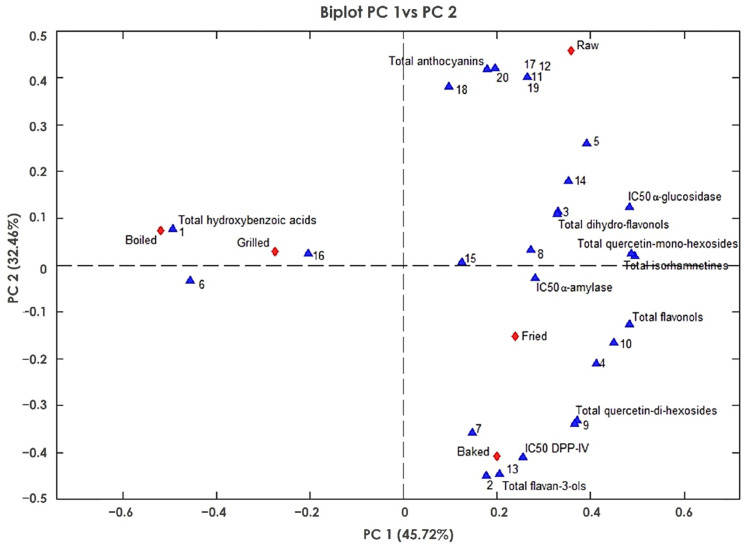
Principal component analysis (PCA). Loading plot of PC1 vs. PC2. The code number of compounds is reported in Table 2. The symbol ▲ identifies compounds, while the symbol ♦ identifies different cooking treatments. The individual compounds are reported with the same number as listed in Table 2.

To further understand the relationship between cooking treatments and the biological activities and to identify potential active compounds in red-skinned onion, principal component analysis (PCA) was carried out (Figure 4). Two principal components account for 78.18% of the total variance. The bi-plot PC1 vs. PC2 displayed an evident splitting of the treatments. Boiled and grilled samples had a negative PC1 score and formed the first group, which was dominated by the presence of hydroxybenzoic acids. The other group consisted of the raw, baked, and fried samples, which were positively related to PC1. PC2 was mainly associated with different classes of compounds and had a positive loading for total hydroxybenzoic acids, total anthocyanins, total dihydro-flavonols, and total isorhamnetins, while total flavonols and flavan-3-ols had a negative loading value. 

By focusing on PC1 and PC2, it is possible to identify the most active compounds and thus discriminate between different heat treatments. Baked and fried samples had a negative score on PC2 and were positively correlated with the IC_50_ of DPP-IV. In addition, a positive correlation between the IC_50_ of DPP-IV and the presence of quercetin-di-hexosides was observed. In contrast, raw, baked and fried samples, which had a positive score on PC1, were positively correlated with the IC_50_ of α-glucosidase and the amount of quercetin-mono-hexosides and total isorhamnetins. Finally, no clear relationship was observed between the α-amylase inhibition and specific classes of compounds.

#### 3.4.2. Correlation and Linear Regression Analysis

**Figure 5:**
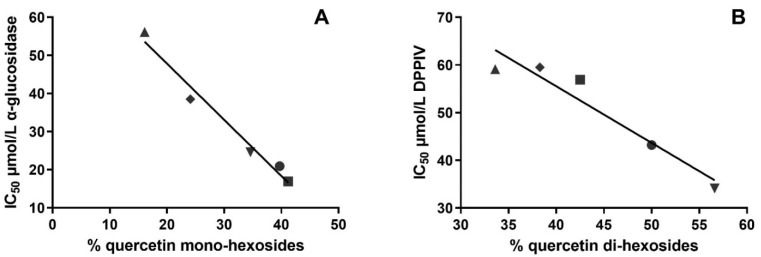
Linear regression analysis. (**A**) Relationship between the content of quercetin mono-hexosides in red-skinned onion samples and the IC50 values against the enzyme α-glucosidase. (**B**) Relationship between the content of quercetin di-hexosides in red-skinned onion samples and the IC50 against the enzyme di-peptidyl-peptidase-IV (DPP-IV). The symbol ▲ identifies boiled samples, ♦ grilled samples, ● fried samples, ■ raw samples, and ▼ baked samples.

To gain more detailed information about the possible associations among specific classes of compounds and the related inhibitory activity toward the three key enzymes, correlation and linear regression studies were also carried out. As reported in Figure 5A, a statistically significant inverse correlation (*p* < 0.05) between the concentration of quercetin-mono-hexosides in a specific sample and the IC_50_ values against α-glucosidase was observed (R^2^ = 0.9756 and Pearson *r* = −0.9877). No further correlations were found between the α-glucosidase-inhibitory activity and other specific classes of phenolic compounds. These results confirmed the PCA results and strongly identified quercetin-mono-hexosides as the compounds responsible for the α-glucosidase-inhibitory activity in onion samples. Previously, quercetin-4′-*O*-glucoside was identified as the compound responsible for the α-glucosidase-inhibitory activity of hot water extracts of peel from different onion varieties, whereas quercetin-3-*O*-hexoside-4′-*O*-hexoside was not active [51]. In another study, kaempferol-3-*O*-glucoside was found to be an effective inhibitor of α-glucosidase [52]. Moreover, some other investigations generically identified flavonols as the active compounds against α-glucosidase in plant extracts [19]. Previous structure–activity relationship studies highlighted that the catechol moieties in the A- and B-rings as well as the OH group at the C3 position were of paramount importance for the inhibitory activity of flavonoids against α-glucosidase [53,54]. However, the supposed active compounds identified in in vitro digested red-skinned onion were glycosylated at the C4′ and/or C3 position. We can suppose that the hexose moiety present in the structure of these compounds may interact with the active site of the enzyme in analogy with the substrate (maltose).

Correlation and linear regression analysis, carried out considering the IC_50_ values against DPP-IV and the different classes of phenolic compounds identified in the in vitro digested onion samples, showed quercetin-di-hexosides as the principal active compounds against DPP-IV. As depicted in Figure 5B, a strong inverse correlation (R^2^ = 0.9189 and Pearson *r* = −0.9586; *p* < 0.05) was ascertained between quercetin-di-hexosides and IC_50_ values versus the enzyme DPP-IV. Very few studies have been performed regarding the interaction between flavonols and DPP-IV. In a recent article, the authors identified quercetin-3-*O*-glucoside-7-*O*-rhamnoside as a potent inhibitor of DPP-IV [55]. In addition, kaempferol-3-*O*-glucoside-*O*-galactoside was able to inhibit efficiently DPP-IV activity by binding the DPP-IV S1 pocket with the flavone core structure and the DPP-IV S2 pocket with the disaccharide moiety [50]. On the contrary, flavonol-mono-glucosides have been characterized as weak DPP-IV inhibitors [56,57]. All together, these results suggest that quercetin-di-hexosides are potent DPP-IV inhibitors.

Finally, no correlation was found between specific compounds and the α-amylase inhibitory activity of in vitro digested red-skinned onion. Previous studies found that the hydroxylation of flavonoids favored the inhibitory activity, whereas glycosylation decreased it [58]. These data can explain why in vitro digested RSO phenolic-rich extracts were more effective against α-glucosidase with respect to α-amylase.

## 4. Conclusions

This study demonstrates that different heat treatments associated with the in vitro gastrointestinal digestion changed the phenolic composition of phenolic-rich vegetables such as dark purple eggplant and red-skinned onion. In fact, some compounds were more bioaccessible, whereas others were degraded following cooking and in vitro digestion. More interestingly, by tuning the phenolic profile after digestion, heat treatments modulate the bioactive profiles of phenolic-rich vegetables. The results presented in the study proved that all the red-skinned onion samples presented some inhibitory activity against the three key enzymes involved in the diabetes pathogenesis but to a different extent depending on the cooking method and phenolic profile. The class of flavonols is the one that has been found to be most active especially against α-glucosidase and DPP-IV enzymes. Furthermore, statistical analysis confirmed a significant correlation between the amount of quercetin mono-hexosides (mainly quercetin-4′-*O*-hexoside) and the IC_50_ of α-glucosidase and the amount of quercetin di-hexosides (mainly quercetin-3-*O*-hexoside-4′-*O*-hexoside) and the IC_50_ of DPP-IV. This is of paramount importance, as the regular intake of foods rich in flavonols (such as red-skinned onion) could help prevent the metabolic syndrome. In addition, an accurate design of the cooking methods may be pivotal in suggesting the most suitable process to optimize the release of specific phenolic compounds and the alleged bioactivities. However, additionally, in vivo studies are strongly required to corroborate the physiological relevance of the in vitro results, especially related to the cooking impact on phenolic compounds absorption, metabolism, and health effect, which is a field of research not yet sufficiently considered and explored.

## Figures and Tables

**Table 1:** Concentration of phenolic compounds in in vitro digested raw and cooked dark purple eggplant phenolic-rich fractions. Results are reported as μmol of phenolic compounds/100 g of raw or cooked eggplant.

In Vitro Digested Dark Purple Eggplant Phenolic-Rich Fractions					
Compound	Raw	Baked	Boiled	Fried	Grilled
**Hydroxycinnamic Acids**					
Caffeoylshikimic acid	n.d.	n.d.	n.d.	n.d.	1.01 ± 0.08
3-*O*-Caffeoylquinic acid	0.31 ± 0.01 ^c^	8.44 ± 0.03 ^a^	6.03 ± 0.03 ^b^	5.26 ± 0.03 ^b^	8.12 ± 0.97 ^a^
4-*O*-Caffeoylquinic acid	n.d.	3.10 ± 0.11 ^b^	2.47 ± 0.03 ^c^	3.01 ± 0.03 ^b^	3.45 ± 0.04 ^a^
5-*O*-Caffeoylquinic acid *trans*	2.86 ± 0.08 ^d^	30.29 ± 0.50 ^b^	19.25 ± 0.89 ^c^	32.26 ± 0.56 ^a^	33.17 ± 0.99 ^a^
5-*O*-Caffeoylquinic acid *cis*	0.34 ± 0.00 ^e^	10.13 ± 0.13 ^c^	3.25 ± 0.12 ^d^	12.01 ± 0.04 ^b^	14.03 ± 0.58 ^a^
3-*O*-Feruloylquinic acid *cis*	n.d.	0.53 ± 0.00 ^a^	n.d.	n.d.	0.12 ± 0.00 ^b^
3-*O*-Feruloylquinic acid *trans*	n.d.	1.49 ± 0.03 ^b^	n.d.	n.d.	4.09 ± 0.10 ^a^
5-*O*-Feruloylquinic acid *trans*	n.d.	2.08 ± 0.05 ^b^	n.d.	1.22 ± 0.01 ^b^	19.96 ± 1.13 ^a^
4-*O*-Feruloylquinic acid	0.26 ± 0.00 ^b^	0.68 ± 0.02 ^a^	n.d.	n.d.	n.d.
5-*O*-Feruloylquinic acid *cis*	0.63 ± 0.00 ^d^	5.66 ± 0.09 ^b^	n.d.	18.36 ± 0.70 ^a^	2.12 ± 0.03 ^c^
1,5-Di-*O*-caffeoylquinic acid	n.d.	n.d.	n.d.	n.d.	2.23 ± 0.08
3,4-Di-*O*-caffeoylquinic acid	0.56 ± 0.01 ^c^	n.d.	n.d.	17.68 ± 0.08 ^a^	13.16 ± 0.20 ^b^
4,5-Di-*O*-caffeoylquinic acid	n.d.	n.d.	n.d.	29.27 ± 0.86 ^a^	8.58 ± 0.04 ^b^
3,5-Di-*O*-caffeoylquinic acid	n.d.	n.d.	n.d.	24.52 ± 0.36 ^b^	36.13 ± 0.83 ^a^
Caffeoylquinic acid dehydrodimer isomer	1.61 ± 0.04 ^b^	2.79 ± 0.05 ^a^	0.79 ± 0.02 ^d^	0.92 ± 0.02 ^c^	1.65 ± 0.01 ^b^
Caffeoylquinic acid dehydrodimer isomer	1.36 ± 0.04 ^b^	1.34 ± 0.04 ^b^	1.36 ± 0.10 ^b^	n.d.	4.12 ± 0.09 ^a^
Caffeoylquinic acid dehydrodimer isomer	0.99 ± 0.02 ^d^	3.20 ± 0.09 ^b^	1.60 ± 0.08 ^c^	n.d.	5.32 ± 0.06 ^a^
Caffeoylquinic acid dehydrodimer isomer	1.47 ± 0.04 ^c^	5.48 ± 0.14 ^a^	0.70 ± 0.05 ^d^	n.d.	1.89 ± 0.06 ^b^
Caffeoylquinic acid dehydrodimer isomer	n.d.	11.49 ± 0.15 ^a^	1.86 ± 0.11 ^b^	n.d.	2.03 ± 0.07 ^b^
**Eggplant**					
**Compound**	**Raw**	**Baked**	**Boiled**	**Fried**	**Grilled**
Caffeoylquinic acid dehydrodimer isomer	n.d.	n.d.	n.d.	n.d.	0.87 ± 0.01
N^1^,N^5^-Di-caffeoyl-spermidine	n.d.	6.15 ± 0.17	n.d.	n.d.	n.d.
N^1^,N^10^-Di-caffeoyl-spermidine	n.d.	n.d.	n.d.	7.66 ± 0.03	n.d.
N^1^,N^5^-Di-dihydrocaffeoyl-spermidine	n.d.	n.d.	n.d.	107.50 ± 4.02 ^a^	11.58 ± 0.20 ^b^
N^1^,N^10^-Dihydrocaffeoyl-caffeoyl-spermidine	n.d.	n.d.	n.d.	37.40 ± 0.54	n.d.
Total hydroxycinnamic acids	10.39 ± 0.24 ^e^	92.87 ± 1.60 ^c^	37.32 ± 1.44 ^d^	297.07 ± 7.28 ^a^	173.63 ± 5.57 ^b^
**Flavonols**					
Quercetin-di-*O*-hexoside	0.23 ± 0.00	n.d.	n.d.	n.d.	n.d.
Kaempferol-3-*O*-rutinoside	0.11 ± 0.00 ^b^	n.d.	n.d.	0.87 ± 0.02 ^a^	n.d.
Total flavonols	0.35 ± 0.01 ^b^	n.d.	n.d.	0.87 ± 0.02 ^a^	n.d.
Total	10.74 ± 0.25 ^e^	92.87 ± 1.60 ^c^	37.32 ± 1.44 ^d^	297.95 ± 7.29 ^a^	173.63 ± 5.57 ^b^

Different letters within the same row mean significant different (*p* < 0.05) values. n.d. means that the compound was not detected in the sample.

**Table 2:** Concentration of phenolic compounds in in vitro digested raw and cooked red-skinned onion phenolic-rich fractions. Results are reported as μmol of phenolic compounds/100 g of raw or cooked onion.

In Vitro Digested Red-Skinned Onion Phenolic-Rich Fractions						
	Compound	Raw	Baked	Boiled	Fried	Grilled
**Hydroxybenzoic acids**						
1	Protocatechuic acid-*O*-hexoside	n.d.	n.d.	42.80 ± 0.24 ^b^	n.d.	51.35 ± 0.22 ^a^
	Total hydroxybenzoic acids	n.d.	n.d.	42.80 ± 0.24 ^b^	n.d.	51.35 ± 0.22 ^a^
**Flavan-3-ols**						
2	(Epi)catechin-3-*O*-hexoside isomer	n.d.	1.55 ± 0.01 ^a^	n.d.	0.75 ± 0.01 ^c^	1.02 ± 0.00 ^b^
	Total flavan-3-ols	n.d.	1.55 ± 0.01 ^a^	n.d.	0.75 ± 0.01 ^c^	1.02 ± 0.00 ^b^
**Dihydro-flavonols**						
3	Taxifolin-*O*-hexoside isomer	0.09 ± 0.00 ^b^	n.d.	n.d.	0.15 ± 0.00 ^a^	n.d.
	Total dihydro-flavonols	0.09 ± 0.00 ^b^	n.d.	n.d.	0.15 ± 0.00 ^a^	n.d.
**Flavonols**						
4	Quercetin-3-*O*-hexoside isomer	11.51 ± 0.08 ^d^	18.70 ± 0.07 ^b^	n.d.	15.17 ± 0.07 ^c^	20.69 ± 0.06 ^a^
5	Quercetin-4′-*O*-hexoside	22.76 ± 0.11 ^a^	12.90 ± 0.10 ^d^	10.27 ± 0.01 ^e^	18.02 ± 0.07 ^b^	15.94 ± 0.01 ^c^
6	Quercetin-3-*O*-glucoside	0.91 ± 0.01 ^d^	1.72 ± 0.02 ^b^	3.81 ± 0.08 ^a^	1.42 ± 0.01 ^c^	3.71 ± 0.05 ^a^
7	Quercetin-3-*O*-hexoside-7-*O*-hexoside	n.d.	1.83 ± 0.00	n.d.	n.d.	n.d.
8	Quercetin-7-*O*-hexoside-4′-*O*-hexoside	1.78 ± 0.00 ^c^	2.12 ± 0.01 ^a^	n.d.	n.d.	1.83 ± 0.00 ^b^
9	Quercetin-3-*O*-hexoside-4′-*O*-hexoside	34.36 ± 0.10 ^d^	50.49 ± 0.10 ^b^	29.45 ± 0.05 ^e^	43.54 ± 0.16 ^c^	62.08 ± 0.81 ^a^
10	Quercetin-tri-*O*-hexoside isomer	0.92 ± 0.01 ^c^	1.29 ± 0.00 ^a^	0.21 ± 0.00 ^d^	0.94 ± 0.01 ^b^	1.28 ± 0.00 ^a^
11	Kaempferol-7-*O*-hexoside isomer	0.13 ± 0.00	n.d.	n.d.	n.d.	n.d.
12	Kaempferol-3-*O*-hexoside isomer	0.23 ± 0.0	n.d.	n.d.	n.d.	n.d.
13	Isorhamnetin-3-*O*-hexoside isomer	n.d.	2.33 ± 0.01 ^a^	n.d.	1.73 ± 0.00 ^b^	1.60 ± 0.00 ^c^
14	Isorhamnetin-4′-*O*-hexoside	4.45 ± 0.04 ^b^	n.d.	n.d.	5.37 ± 0.06 ^a^	n.d.
15	Isorhamnetin-3-*O*-hexoside-4′-*O*-hexoside	2.37 ± 0.00 ^c^	3.28 ± 0.01 ^b^	1.04 ± 0.01 ^d^	n.d	3.35 ± 0.00 ^a^
	Total flavonols	79.42 ± 0.36 ^d^	94.66 ± 0.33 ^b^	44.78 ± 0.15 ^e^	86.19 ± 0.37 ^c^	110.49 ± 1.08 ^a^
**Anthocyanins**						
16	Cyanidin-3-*O*-hexoside	n.d.	n.d.	n.d.	n.d.	0.73 ± 0.00
**Red-skinned onion**						
	**Compound**	**Raw**	**Baked**	**Boiled**	**Fried**	**Grilled**
17	Cyanidin-*O*-malonyl-hexoside isomer	2.02 ± 0.02	n.d.	n.d.	n.d.	n.d.
18	Cyanidin-*O*-hexoside-*O*-hexoside isomer	1.24 ± 0.00 ^b^	n.d.	n.d.	n.d.	1.90 ± 0.01 ^a^
19	Cyanidin-*O*-hexoside-*O*-hexoside isomer	0.79 ± 0.00	n.d.	n.d.	n.d.	n.d.
20	Cyanidin-*O*-hexoside-*O*-malonyl-hexoside isomer	1.92 ± 0.00 ^a^	n.d.	n.d.	n.d.	1.62 ± 0.01 ^b^
	Total anthocyanins	5.98 ± 0.03 ^a^	n.d.	n.d.	n.d.	4.25 ± 0.02 ^b^
	Total	85.49 ± 0.39 ^d^	96.21 ± 0.34 ^b^	87.58 ± 0.39 ^c^	87.09 ± 0.38 ^c,d^	167.11 ± 1.18 ^a^

Different letters within the same row mean significant different (*p* < 0.05) values. n.d. means that the compound was not detected in the sample.

## Data Availability

The data presented in this study are available in here.
